# Genomic Dissection of Chinese Yangtze River Delta White Goat Based on Whole Genome Sequencing

**DOI:** 10.3390/ani15070979

**Published:** 2025-03-28

**Authors:** Jun Gao, Lingwei Sun, Rongrong Liao, Yuhua Lyu, Shushan Zhang, Jiehuan Xu, Mengqian He, Caifeng Wu, Defu Zhang, Yuexia Lin, Jianjun Dai

**Affiliations:** 1 Institute of Animal Science and Veterinary Medicine, Shanghai Academy of Agricultural Sciences, Shanghai 201106, China; gaojun@saas.sh.cn (J.G.); sunlingwei1987@126.com (L.S.); lrrnd@163.com (R.L.); 18918162132@163.com (Y.L.); smalltreexj@126.com (S.Z.); jiehuanxu810@163.com (J.X.); he1037247863@163.com (M.H.); wucaifengwcf@163.com (C.W.); zhangdefuzdf@163.com (D.Z.); 2 Shanghai Municipal Key Laboratory of Agri-Genetics and Breeding, Shanghai 201106, China; 3 Key Laboratory of Livestock and Poultry Resources Evaluation and Utilization, Ministry of Agriculture and Rural Affairs, Shanghai 201106, China

**Keywords:** Chinese Yangtze River Delta white goat, whole genome sequencing, selective sweep analysis

## Abstract

The conservation and utilization of livestock genetic resources is essential for the maintenance of biodiversity and breed innovation. Whole genome sequencing (WGS) was performed on 90 samples from Chinese Yangtze River Delta White goats (YRD), sourced from two populations of Chongming island white goats and Haimen white goats, aiming to dissect their genomic characteristics. In addition, 262 WGS data from nine other breeds of goats were downloaded from the NCBI database. These WGS data obtained were used to identify and analyze genetic variation with the goat reference genome, and the genetic structure of goat populations was analyzed. Through selective sweep analysis, the selection-signature genes and their polymorphic features were identified. It was found that the most significant genomic selection region in YRD goats was in the region of 62.9–64.6 Mb on chromosome 13, which contained genes related to the coat color and muscle growth of the goats. Nucleotide diversity of MYH7B, a gene related to the development of the goat’s skeletal muscle, within the Yangtze River Delta white goat population was significantly lower than in other domestic and foreign goat breeds, suggesting that the gene was subject to selection. In addition, the IGF2BP2 gene, reported to be associated with litter size in goats, showed clear selection-signature characteristics in the Boer goats compared to the YRD goats.

## Simple Summary

Whole genome sequencing was performed on the Chinese Yangtze River Delta White goat, aiming to analyze their population genetic structure and genomic characteristics. Selective sweep analysis identified selection-signature genes and their polymorphisms in the goat populations.

## 1. Introduction

The Yangtze River Delta white goat (YRD) is an indigenous goat breed in China, which has been included in the Chinese National List of Livestock and Poultry Genetic Resources for Conservation and in the United Nations Food and Agriculture Organization’s Domestic Animal Diversity Information System (DAD-IS) [1]. This breed is known for its roughage tolerance, high fertility, and excellent meat quality. Although the mutton products of this breed have a good reputation for added value in the production area market, compared to some international specialized meat goat breeds (e.g., Boer goat), the size of the YRD goat is small and its meat-producing capacity is also relatively low; mature weights for bucks and does are 23–32 and 18–25 kg, respectively [2]. However, this area of study has lagged behind in terms of both sample size and sequencing methodology. Thus, how to improve the growth and meat-producing performance of YRD goats has always been a concern in the selection and breeding of this breed. In addition, the YRD goat is a suitable animal model for experimental use because of its relatively small size and ease of husbandry. It has been used as a goat model for acute spinal cord compression injury research [3].

It is generally recognized that there are two main populations of YRD goats; one is distributed in the Chongming Island area of Shanghai in China and is known as the Chongming white goat (CM), and the other population is distributed in the Haimen area of the Jiangsu Province in China and is known as the Haimen goat (HM). Both areas belong to the Yangtze River Delta region of China, so the two groups are collectively referred to as the YRD goats. To the best of our knowledge, individual sequencing data for YRD breeds with sufficient numbers is not currently available in public databases, so this study aims to fill this gap and also provide an analyzable source of data on goat breeds in East Asia for global goat breed research. The only previous report in this area is a study by the authors of this paper in 2020 based on the resequencing of a mixed pool of the Chongming white goat group [2]. With the development of high-throughput sequencing technology and the associated reduction in sequencing costs, we believe that it is necessary to conduct a larger-scale sequencing study of YRD goats for the genomics study. This will improve the representativeness of the YRD goat population and systematically reveal the population genetic structure, selection signatures, and phenotypic associations.

To detect “selection signatures” in the genome, a series of statistical methods called the “selective sweep” has been proposed, comprising such factors as the genetic differentiation value (F_ST_) [4], polymorphism statistic (θπ) [5], and integrated haplotype homology score (iHS) [6], etc. Through the analysis of selection signatures, we will study the functional genes related to the growth and reproductive performance of goats, thus providing the possibility for breeding improvement in YRD goats. The joint analysis of selective sweep methods can improve the accuracy of the selection signatures. These methods, such as the F_ST_ and θπ intersection methods, used in this study for selection-signature dissection have been successfully applied in other goat genomic studies [7,8].

In this study, we performed whole-genome sequencing on the above mentioned YRD goat populations. Through genome-wide genetic variation identification and genetic structure analysis, we aimed to dissect the genomic characteristics of this goat breed, and to explore the genes with selection signatures with other foreign and Chinese goat breeds.

## 2. Materials and Methods

### 2.1. Sample Collection

The blood of 60 unrelated CM goat individuals was collected from Shanghai YuanDu White Goat Breeding Farm (designated breeding farm; approximate stocking size is 1200 heads, Shanghai, China). The blood of 30 unrelated HM goat individuals was collected from Haimen JinSheng Goat Farm (designated breeding farm, Haimen, Jiangsu Province, China). The samples covered all recorded pedigrees, and exhibited a male to female ratio of 1:2. Blood samples were collected from the jugular veins of goats using a sterile blood sampler with a volume of 2–3 mL per goat. The collection tubes were commercial blood collection tubes and contained EDTA anticoagulant.

### 2.2. Genomic DNA Extraction and Sequencing Libraries Construction

In this study, goat genomic DNA was extracted from the anticoagulated whole blood of goats using the conventional phenol-chloroform method [9]. The quantity of DNA was measured with a NanoDrop (Thermo Fisher Scientific, MA, USA), and the quality was checked via agarose electrophoresis. NGS library preparation kits were provided by MolBreeding Biotechnology (Shijiazhuang, China). The process included DNA fragmentation, end repair, A-tailing ligation, adapter ligation, and PCR amplification.

### 2.3. High-Throughput Sequencing and Other Sequencing Data Downloads

Sequencing was performed using the MGI-2000 sequencing platform (BGI, Shenzhen, China), and the sequencing mode was PE150 mode. A further 262 sets of WGS data from nine goat breed populations was downloaded from the NCBI database (https://www.ncbi.nlm.nih.gov/, accessed on 5 June 2024). This information can be found in the Supplementary Data S1.

### 2.4. Mapping and Variant Calling

Raw reads obtained by high-throughput sequencing were filtered using fastp v0.20 [10] and then mapped to the ARS1 goat reference genome (GCF_001704415.1, from the NCBI database) [11] using Burrows-Wheeler Aligner (BWA) v0.7.17 software [12]. The GATK (V4.0.3.0) was used for variant calling [12]. In order to obtain high-quality variants, a filter command for the VariantFiltration set was applied with “QD < 2.0||QUAL < 30.0||MQ < 40.0||FS > 60.0||SOR > 3.0||MQRankSum < 12.5||ReadPosRankSum < −8.0”. Further, variant annotations were performed using ANNOVAR (version 2019 Oct 24) [13], and the gene annotation file was downloaded from the Ensembl database (https://asia.ensembl.org/index.html, accessed on 22 November 2024).

### 2.5. Population Genetic Analysis

Population genetic structure analysis was performed after filtering SNPs on goat autosomes using Plink v1.9 [14] software with the following filters: --mind 0.1, --maf 0.05, --hwe 0.000001, and LD pruning with --indep-pairwise 100 50 0.2. The principal component analysis of the population was calculated and plotted using PLINK v1.9 and R package [14]. ADMIXTURE v1.3.0 [15] was used to infer the optimal number of ancestral clusters K, which were tested and ranged from 2 to 15. The phylogenetic tree was built based on the IBS distance matrix using vcf2phylip-2.0 and FastME 2.0 [16], and visualized by iTOL v6 [17].

### 2.6. Genome-Wide Selective Sweeps Detection

We aimed to explore the potential selection signature at the genomic level in the YRD population (grouped as 90 individuals from two populations: CM and HM) and other goat populations (grouped as 262 individuals from nine breeds, including Boer, Saannen, Angora, etc.). The F_ST_ and θπ ratios on the genome were calculated using the crossover method, using a window with a step size of 100 kb, and aligning the genome window to match the top 5% of the F_ST_. The top 5% of the overlapping translation window was taken as the θπ ratio (YRD/Others) and as a candidate selection region, and bcftools v1.10.2 [18] and gene annotation files were used to extract subsequences to study the polymorphism levels of the candidate genes.

### 2.7. Enrichment Analysis and PPI Network Construction

To further explore the potential biological significance of genes within these candidate regions, Gene Ontology (GO) terms and Kyoto Encyclopedia of Genes and Genomes (KEGG) pathway enrichment analyses were carried out using the universal enrichment tool clusterProfiler v4.8.1 [19]. Next, to further investigate the interaction associations of selection-signature genes, we applied the selection-signature genes to the STRING (Search Tool for the Retrieval of Interacting Genes/Proteins) database (https://string-db.org/, accessed on 8 December 2024) [20] for PPI (Protein–Protein Interaction) analysis. The selection-signature genes were mapped and the interactions with a default confidence cutoff of 0.4 were used. Afterward, a protein–protein interaction (PPI) network was constructed, and Markov Cluster Algorithm (MCL) clustering was used for subnetwork construction, in which the inflation parameter used a default setting of 3.

## 3. Results

### 3.1. Genetic Variant Identification

Whole-genome sequencing of 90 individuals from two YRD populations (CM, HM) generated a total size of 2.46 Tb data, which has been deposited in the publicly accessible website: http://www.cncb.ac.cn (China National Center for Bioinformation, CNCB, accessed on 20 December 2024) [21,22] with BioProject designations of PRJCA031876 and PRJCA031233, and GSA numbers of CRA020146 and CRA019905. To fully explore the genomic characterization of the YRD goat, whole genome sequencing data of another nine breeds, comprising 262 goat samples (Supplementary Data S1), were downloaded from a publicly available database. After quality control, the average Q20% and Q30% of YRD samples reached 97.78% and 92.84% (Table 1).

Next, the clean reads were aligned against the ARS1 reference genome using BWA v0.7.17. A total of 54,391,809 SNPs and 12,085,405 InDels were identified. Statistics based on results annotated by ANNOVA (Table 2) showed that the intergenic variants accounted for 62.6% and intronic variants accounted for 27.1% of the total variants.

### 3.2. Population Genetic Structure

**Figure 1:**
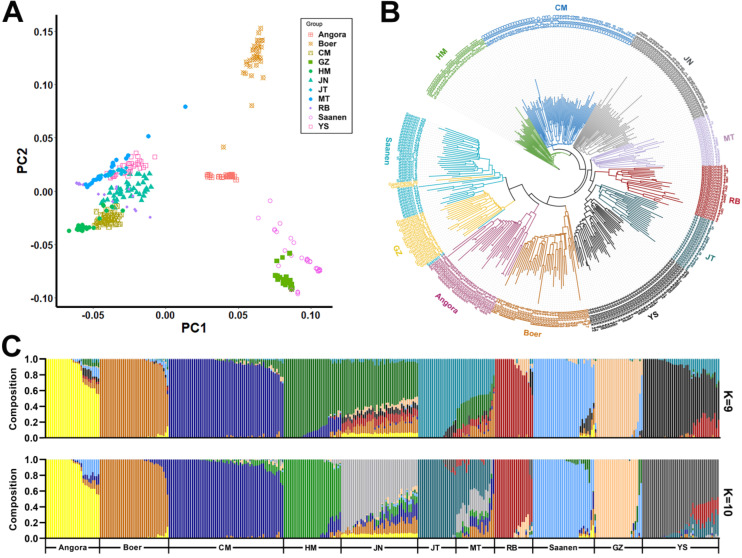
Genetic structure of 11 goat populations. (**A**) PCA analysis of 11 of goat breeds. (**B**) Neighboring tree of 351 goat individuals from 11 populations. (**C**) Genetic structure (K = 9 and 10, with the lowest CV error rate) inferred by admixture analysis.

After multiple screenings, a total of 351 goat individuals and 569,301 SNPs were retained for genetic structure analysis. The PCA results presented a clear differential clustering of Chinese goat and foreign breeds (Figure 1A). Most of the Chinese goat breeds (CM, HM, JN, JT, MT, RB, and YS) were gathered together, while three foreign goat breeds (Boer, Angora, and Saanen) clustered singly. In addition, the GZ dairy goats clustered close to the Saanen goats, revealing that the genomic composition of GZ is considered to be closer to Saanen dairy goats as compared to domestic native goats, which was correspondingly corroborated by the results of the phylogenetic tree (Figure 1B).

The most plausible population structure, inferred based on the admixture structure analysis (Figure 1C) with the lowest cross-validation error values of 0.4869 (K = 9) and 0.4870 (K = 10), was obtained. It was found that CM and HM varieties within the YRD population were still genetically differentiated in genetic structure, although there was a small amount of mutual genetic background infiltration between these two populations. The other two Chinese goat breeds, the Jining goat (JN) from Shandong Province and the Matou goat (MT) from Hubei Province, had more obvious genetic backgrounds of other goat breeds mixed in.

### 3.3. Selection-Signature Detection

A total of 23,213 genomic sliding windows were obtained on the goat genome in this study. The results of this study showed that 342 genomic regions under selection were identified in the YRD (CM and HM) population (the same F_ST_ threshold and θπ (Others)/θπ (YRD) ratio > 1.402841), whereas 118 genomic regions under selection pressure were identified in the Others population (threshold, 5%; F_ST_ > 0.090930 and θπ (YRD)/θπ (Others) ratio > 1.007396).

### 3.4. Functional Analysis of Genes in Selection-Signature Regions

**Figure 2:**
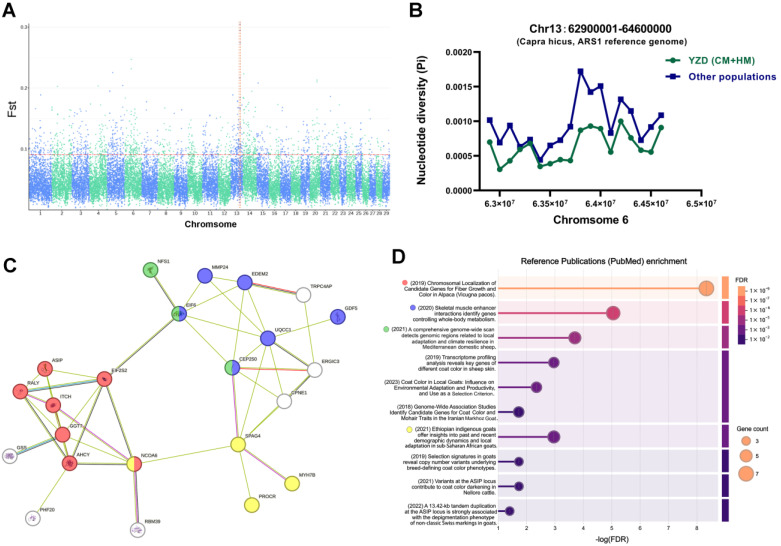
Functional analysis of genes within the selection-signature region of the YRD goat. (**A**) The most differentiated signal of F_ST_ occurs on chromosome 13. (**B**) Nucleotide polymorphisms were significantly reduced in YRD goats within the span of 62.9−64.6 Mb on chromosome 13. (**C**,**D**). Protein–protein interaction and reference publication enrichment analysis showed that genes within the region were significantly associated with coat color, skeletal muscle, and environmental adaptation in goats.

A total of 626 selection−signature genes were identified in the YRD goats, and 175 selection−signature genes were identified in the Others population (Supplementary Data S2). The most significant selection-signature region on the YRD goat genome is located on chromosome 13 in the 62.9–64.6 Mb region, which has the most pronounced Fst differentiation signal (Figure 2A) and significant differences in nucleic acid diversity (Figure 2B).

PPI analysis (Figure 2C) of the selection−signature genes in this region and enrichment of the literature (Figure 2D) showed that some of these selection-signature functional genes are closely related to goat coat color (e.g., ASIP, AHCY, RALY), while others are related to goat skeletal muscle development (e.g., MYH7B, GDF5, and UQCC1), and others may be related to goat adaptation (ELF6, NFS1).

### 3.5. Polymorphisms of MYH7B Within YRD Population

**Figure 3:**
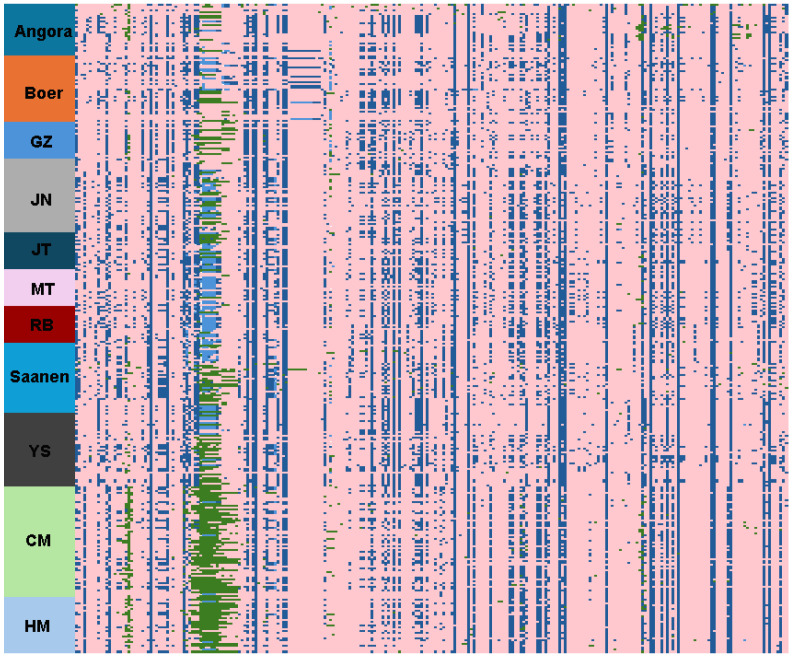
Gene polymorphisms of MYH7B in goat populations. In Figure 3, the reference genome is indicated in pink (0/0), the blue parts indicate the loci of genotypes (0/1 and 1/1), and the green parts represent the deletion genotype (./.).

MYH7B is located at position 63851003–63875038 on chromosome 13 (NC_030820.1) of the ARS1 goat reference genome. A total of 313 variants were detected within the MYH7B gene of the 352 goat individuals in this study. A genotype heatmap of the MYH7B was constructed by combining these 313 variants as columns and the sample individuals as rows (Figure 3). Detailed polymorphism information can be found in Supplementary Data S2.

MYH7B in YRD goats demonstrates more consistent sequence features, such as having more site deletion genotypes (./.) at 63855870–63855971 (Figure 3, green marking), in addition to the overall polymorphism of the gene being reduced compared to the other goat breeds, indicating that the gene has been subjected to significant selection in the YRD population and that the physical location of this gene is within the segment with the most significant differences in nucleotide polymorphisms (Figure 2B).

### 3.6. IGF2BP2 Under Selection in Boer Goat

The YRD breed is of relatively small size, but exhibits excellent litter performance. Its counterpart, the Boer goat, is recognized worldwide as a breed with excellent growth but poor litter performance. Selection-signature dissection also compared YRD goats individually with Boer goats. Therefore, this study also identified 350 selection-signature genes, such as IGF2BP2, in Boer goats (Supplementary Data S2).

**Figure 4:**
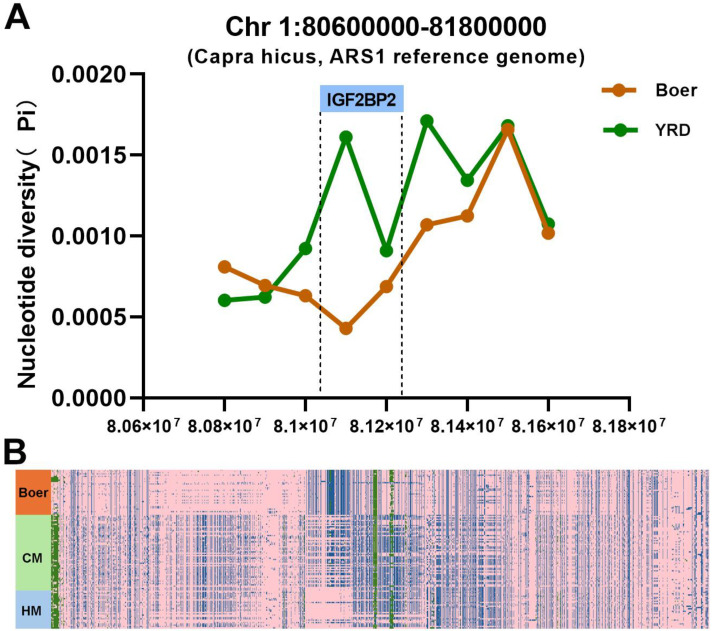
Selection signatures of the IGF2BP2 gene between Boer and YRD goats. (**A**) Nucleotide polymorphisms in IGF2BP2 in the Boer goat compared to the YRD population. (**B**) The genotype polymorphisms of IGF2BP2 in the YRD population and Boer breed. Consistent with the reference genome indicated in pink (0/0), the blue parts indicating the loci of genotypes (0/1 and 1/1), and the green parts representing the deletion genotype (./.).

IGF2BP2 is located at position 81081005–81245002 on chromosome 1 (NC_030808.1) of the ARS1 goat reference genome. The polymorphism of IGF2BP2 was found to be significantly lower in the Boer breed than in the YRD goats (Figure 4A), and the 2092 variants of genotype polymorphisms in IGF2BP2 were significantly different between the YRD and Boer goats (Figure 4B).

## 4. Discussion

Chinese indigenous goat breeds have rich genetic diversity and are an important part of the world’s goat genetic resources. The YRD goat, as a representative breed of Chinese indigenous goats, has been listed in the Chinese National List of Livestock Genetic Resources Conservation, but its genetic traits and genomic characterization have not yet been fully studied. The present study is the largest sequencing study of this breed known to date and may provide an accumulation of data for future studies on the origin, differentiation, and migration of goats in East Asia.

From the phylogenetic tree and genetic structure determined in this study, YRD goats are evolutionarily closer to Jining goats distributed in Jining area of Shandong Province. This point is also consistent with the correlation between the degree of population differentiation and geographical distance in terms of phylogeny. The Jining goat population also showed obvious signs of infiltration of genetic components from the YRD goats, and HM and JN were merged into one group when K = 9, suggesting that Haimen goats and Jining grey goats have more genomic similarities. It is when K = 10 that the JN breed can be separated from the HM breed. In contrast, two breeds of CM and HM, which belong to the same YRD population, are genetically differentiated to some extent. Although there is a small amount of genetic component infiltration across both groups, they do not appear in each other’s evolutionary branches in terms of individual mixing. There are other WGS-based studies [23] on dairy goats that have shown that GZ are crossbred breeds with short breeding times, whose fathers are Saanen. This is consistent with our results; we found that the GZ and Saanen goats had a close genetic relationship and similar genetic backgrounds, aligning with the history of the formation of these breeds.

This study explored genomic regions and associated genes with demonstrated selection signatures in the YRD population. The most notable region is the 62.9–64.6 Mb region located on chromosome 13, which has the most pronounced Fst differentiation signal and significant differences in nucleic acid diversity. One of the locations with the most pronounced signals is MYH7B (myosin heavy chain 7B). MYH7B transcripts have been confirmed in several conventional mammalian muscles, including the heart, soleus, tibialis anterior, quadriceps, and diaphragm [24,25]. The sarcomeric myosin gene, Myh7b, encodes an intronic microRNA, miR-499, which regulates cardiac and skeletal muscle biology [26]. MyoD and the lymphoid transcription factor Ikaros 4 (Eos) form an active transcriptional complex on the chromatin to regulate the expression of the endogenous Myh7b/miR-499 gene in muscle cells [26]. Several slow muscle protein-coding genes, including MYH7B, are expressed at significantly higher levels in layers than in broilers, which may contribute to the slower growth rate of muscle cells [27]. The effect and mechanism of MYH7B on skeletal muscle development in goats remains to be further investigated and elucidated.

Through PPI and literature enrichment analysis, we found that in addition to MYH7B, there are several other genes related to skeletal muscle enhancement in the selection region (e.g., EDEM2, GDF5, and UQCC1) [28]. These genes are related to skeletal muscle development and the formation of the regulatory network for the growth of goats and meat production performance. Several other selection genes related to goat coat color were also detected in this study, such as ASIP, RALY, and AHCY, which have been reported in several studies [29,30]. The RALY-ELF2S2-ASIP locus was reported to have an influence on skin and hair pigmentation in the Chinese Nanjiang yellow goat [31]. ASIP has also been studied as a strong candidate gene associated with black coat color in Chinese goats, and RALY is considered to be associated with saddle tan and black-and-tan phenotypes [32,33].

In comparison between YRD and Boer goats, the study found that the IGF2BP2 gene was characterized by selection, and polymorphisms in this gene were significantly reduced in the Boer goat, which is known for its high growth performance and low fecundity. IGF2BP2 was found to be subjected to selection signatures in whole genome sequencing-based selection traits for litter size in Chinese Dazu black goats [34]. Further, insertion/deletion variants within the IGF2BP2 gene identified in the reported genome-wide selective sweep analysis revealed a correlation with goat litter size [35]. In this study, we found that the polymorphism of the IGF2BP2 gene in Boer goats differed significantly from that of the YRD population. The YRD goat, as an indigenous breed in China, has been characterized by high litter sizes compared to the Boer goat. Our team has been working on the crossbreeding of Boer and CM goats for a long time, hoping to create a new crossbreed with the high growth characteristics of Boer and the high litter size of YRD breed. Therefore, IGF2BP2 is an important candidate gene whose effect on growth and reproductive performance in goat breeding is of interest.

## 5. Conclusions

In this study, whole genome sequencing was performed on two breeds of YRD goats in China. The population genetic structure and genome characterized by selection signatures of YRD goats were resolved by identifying and analyzing genetic variation compared to nine other breeds. The YRD goat population was found to be characterized by a clear selection signature on chromosome 13, and several genes in the segment, including MYH7B, were closely associated with skeletal muscle development. The IGF2BP2 gene on chromosome 1, on the other hand, also had significant nucleotide polymorphism differences in the YRD goat population as compared to Boer goats, which exhibit superior growth performance. The present study provides molecular genetic data and insights for better conservation and utilization of Chinese indigenous YRD goats.

## Figures and Tables

**Table 1:** Quality control statistics of 352 goats. WGS data from 11 breeds of goat.

Breed (Individuals)	Clean Reads Number	Clean Bases (bp)	Q20 (%)	Q30 (%)
CM (60)	182,979,278	27,392,763,250	97.93	93.30
HM (30)	181,124,770	27,126,731,221	97.48	91.91
Angora (28)	248,429,018	37,297,824,732	97.00	92.12
Boer (36)	463,540,265	64,152,217,674	93.85	86.17
Saanen (38)	413,572,169	53,331,587,696	95.83	89.36
Guanzhong (20)	204,353,917	30,555,062,219	97.45	92.74
Jining (40)	226,665,695	33,837,615,989	97.44	95.07
Jingtang (20)	139,666,239	20,923,429,866	97.31	92.34
Matou (20)	239,152,704	35,849,687,942	97.36	92.88
Redbone (20)	356,169,599	35,597,422,417	97.97	91.12
Yunshang(40)	405,412,921	60,633,301,251	98.03	94.20

Note: Data and percentages in the table are average statistics for each breed.

**Table 2:** ANNOVA genetic variation annotation results.

Annotation	SNP	InDel
intergenic	34,911,798	6,701,733
intronic	14,940,079	3,092,147
ncRNA_intronic	2,177,750	425,960
upstream	773,104	181,920
downstream	749,689	179,338
exonic	497,326	43,198
UTR3	178,363	45,921
ncRNA_exonic	84,879	15,518
UTR5	44,842	12,067
upstream;downstream	30,045	8448
splicing	3037	4348
ncRNA_splicing	409	58
exonic;splicing	328	159
UTR5;UTR3	132	28
ncRNA_exonic;splicing	28	9

## Data Availability

The WGS data were deposited in the publicly accessible website: http://www.cncb.ac.cn (China National Center for Bioinformation, CNCB, accessed on 20 December 2024), with BioProject references PRJCA031876 and PRJCA031233 and GSA numbers CRA020146 and CRA019905.

## Supplementary Materials

The following supporting information can be downloaded at: https://www.mdpi.com/article/10.3390/ani15070979/s1, Supplementary Data S1. 262 Goat WGS data downloaded from NCBI database. Supplementary Data S2. Selection signal gene list in YRD and Others population.
